# Draft genome sequence of *Kluyvera ascorbata* HAK22 isolated from human feces

**DOI:** 10.1128/mra.00890-23

**Published:** 2023-12-20

**Authors:** Asha Yadav, Pratik Balwant Shinde, Mahesh S. Dhar, Kalaiarasan Ponnusamy, Robin Marwal, Radhakrishnan V. S., Krishna Kant Sharma

**Affiliations:** 1 Laboratory of Enzymology and Gut microbiology, Department of Microbiology, Maharshi Dayanand University, Rohtak, Haryana, India; 2 Department of Biotechnology, National Centre for Disease Control, Delhi, India; The University of Arizona, Tucson, Arizona, USA

**Keywords:** *Kluyvera ascorbata*, whole genome sequence, antibiotic resistance, pathogen

## Abstract

The whole genome sequence of rare human pathogen *Kluyvera ascorbata* strain HAK22 is reported. The *K. ascorbata* HAK22 was isolated from healthy human from Gurugram, Haryana, India. The draft genome has a length of 4.7 Mbp, with 54.36% GC content and 4,411 proteins, 4,470 genes, and 18 antimicrobial resistance genes.

## ANNOUNCEMENT

*Kluyvera ascorbata* is a Gram-negative coliform of the family Enterobacteriaceae that causes rare human infections. It is reported in various infections including bacteremia, septicemia (1), diarrhea (2), pneumonia, peritonitis, and neonatal meningitis that can be fatal (3, 4).

In the current study, *K. ascorbata* HAK22 was isolated from the feces of antibiotic naïve healthy Indian male, dated 02 November 2021, aged 28 years with BMI (25 kg/m^2^) from Gurugram, Haryana, India. The *K. ascorbata* strain was isolated on the *Yersinia* selective agar (YSA) (HiMedia, M843F) as pink round colonies using 1 mg fecal sample diluted in phosphate-buffered saline. The 0.1 mL of the 10^−2^ dilution was spread on YSA and incubated at 37°C in an aerobic incubator under static condition. The microscopic examination shows Gram-negative rods. The biochemical characterization was denoted as nitrate positive, oxidase negative, indole positive, and citrate positive (4). The antimicrobial susceptibility checked for different antibiotics shows resistance to broad spectrum antibiotics ampicillin, amoxicillin, and cephalosporins (Cefuroxime, Cloxacillin, Ceftriaxone, and Ticarcillin). The antimicrobial susceptibility was determined by standard disk diffusion method using HiMedia antibiotic disks according to Clinical and Laboratory Standards Institute guidelines (5, 6).

A single bacterial colony was inoculated in the nutrient broth for overnight at 37°C. The overnight grown culture was then processed for DNA extraction using phenol-chloroform method. The DNA was quantified using ThermoScientific nanodrop 2000/2000c. Genomic libraries were prepared using Illumina DNA Prep protocol with bead-based normalization. A paired-end whole genome sequencing was performed on an Illumina MiSeq. The sequenced reads were quality filtered using FastQC and trimmed for the removal of low quality reads, adapter, and primer sequence using Trimmomatic v0.32 (7), resulting in 2,125,937 read pairs of 133 bp length totaling to ~276 Mbp with a 54.36% GC content and a 90.64 mean coverage. The whole genome of the isolate was assembled using SPAdes v3.15.5 (8) “-isolate” command yielding a draft genome of 4.7 Mbp, with 70 contigs of length above 500 bp and largest contig of length 298,144 bp (N50; 171,489 bp). The assembly quality was checked by CheckM v1.2.2 (9) and revealed 99.69% completeness. The taxonomy analysis was performed using 16s rRNA gene aligned against SILVA database using BLAST+ v2.14.1, and Type (Strain) Genome Server (https://tygs.dsmz.de/) that resulted in *K. ascorbata*. The NCBI reference genome *K. ascorbata* strain TP1631 (Accession No. NZ_AP022665.1) was used as reference genome for downstream analysis that showed 88.15% mapping identity. The assembly uploaded on GenBank was annotated using NCBI PGAP (10), and PROKKA v1.14.5 (11) was used to identify 16s rRNA gene for further analysis. Gene prediction revealed 4,411 proteins, 4,470 genes, 5 rRNAs, 53 tRNAs, and 1 tmRNA. Further, 18 antimicrobial resistance (AMR) genes were identified when contigs were passed through CARD-RGI v6.0.2 tool (12). The default parameters were used for all software unless otherwise specified.

The reported draft genome sequence of *K. ascorbata* HAK22 will help us to improve our knowledge about this poorly studied Enterobacteria having clinical significance. The whole genome helps us to identify different AMR genes that provide a platform to illustrate various disease causing mechanism and their pathogenicity.

## Data Availability

This Whole Genome Shotgun project has been deposited at DDBJ/ENA/GenBank under the accession JAVLTS000000000. The version described in this paper is version JAVLTS010000000. The genome sequence data for *Klyuvera ascorbata* HAK22 are accessible through NCBI under BioProject ID: PRJNA1014992 with BioSample accession number SAMN37344948 and the Sequence Read Archive accession number is SRR26234412
